# Implications of Sponge Biodiversity Patterns for the Management of a Marine Reserve in Northern Australia

**DOI:** 10.1371/journal.pone.0141813

**Published:** 2015-11-25

**Authors:** Rachel Przeslawski, Belinda Alvarez, Johnathan Kool, Tom Bridge, M. Julian Caley, Scott Nichol

**Affiliations:** 1 Geoscience Australia, National Earth and Marine Observations Group, GPO Box 378, Canberra, Australia; 2 Museum and Art Gallery of the Northern Territory, GPO Box 4646, Darwin, Australia; 3 ARC Centre of Excellence for Coral Reef Studies, James Cook University, Townsville, Australia; 4 Australian Institute of Marine Science, PMB #3, Townsville MC, Townsville, Australia; Victoria University Wellington, NEW ZEALAND

## Abstract

Marine reserves are becoming progressively more important as anthropogenic impacts continue to increase, but we have little baseline information for most marine environments. In this study, we focus on the Oceanic Shoals Commonwealth Marine Reserve (CMR) in northern Australia, particularly the carbonate banks and terraces of the Sahul Shelf and Van Diemen Rise which have been designated a Key Ecological Feature (KEF). We use a species-level inventory compiled from three marine surveys to the CMR to address several questions relevant to marine management: 1) Are carbonate banks and other raised geomorphic features associated with biodiversity hotspots? 2) Can environmental (depth, substrate hardness, slope) or biogeographic (east vs west) variables help explain local and regional differences in community structure? 3) Do sponge communities differ among individual raised geomorphic features? Approximately 750 sponge specimens were collected in the Oceanic Shoals CMR and assigned to 348 species, of which only 18% included taxonomically described species. Between eastern and western areas of the CMR, there was no difference between sponge species richness or assemblages on raised geomorphic features. Among individual raised geomorphic features, sponge assemblages were significantly different, but species richness was not. Species richness showed no linear relationships with measured environmental factors, but sponge assemblages were weakly associated with several environmental variables including mean depth and mean backscatter (east and west) and mean slope (east only). These patterns of sponge diversity are applied to support the future management and monitoring of this region, particularly noting the importance of spatial scale in biodiversity assessments and associated management strategies.

## Introduction

**Fig 1:**
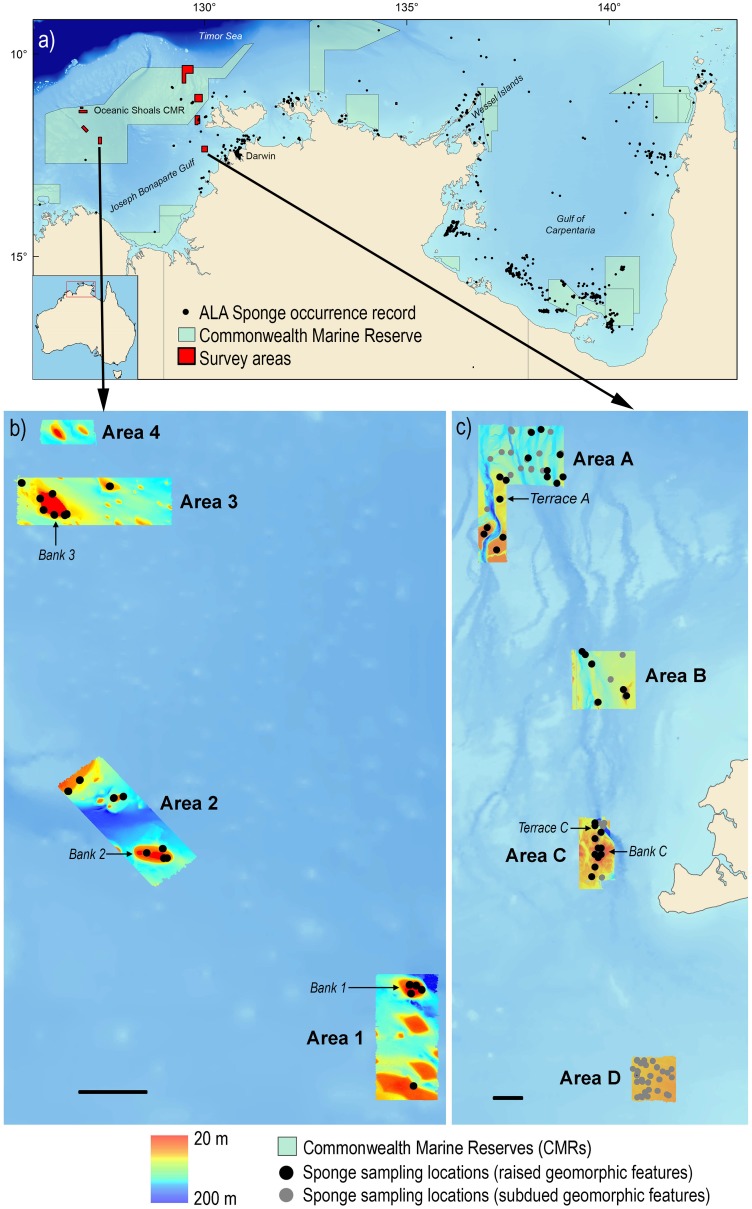
Maps of (a) entire study area, with area mapped by survey area from the current study (red) and sponge occurrence records (black dots) from the Atlas of Living Australia (www.ala.org.au), excluding those collected for the current study, b) western study area of survey SOL5650, and c) eastern study area of surveys SOL4934 and SOL5117. Scale bars represent 10 km. Bathymetry and sampling locations for the opportunistic study area (survey SS2012707) can be found in S1 Fig.

As anthropogenic impacts on the marine environment continue to increase, marine reserves are becoming increasingly important for the management of natural resources [1,2]. Unfortunately, we have little baseline information for most marine environments, particularly for deeper waters [3] or taxa that are difficult to taxonomically identify (e.g. sponges in Fig 1a). Even for well-studied ecosystems such as coral reefs, the available information is often spatially limited and may not match information requirements for effective management [4,5]. As of January 2013, only 28% of Australia to the boundary of the extended continental shelf had been swath-mapped, with just a small proportion of this located in northern Australia [6]. It has also been estimated that only 20–30% of Australian species from non-cryptic macrofaunal groups have been discovered [7], with the number of new, uncertain or undescribed species much higher in deeper waters (e.g. 56% of megabenthic invertebrate species from western Australian margin [8], 95% of crustacean species and 72% of polychaete species [3]). When such little environmental or biological information is available, it is challenging to identify boundaries, appropriate zones, and management strategies for marine reserves [9].

In spite of the challenges related to lack of scientific data, several countries now have large systems of national marine protected areas (e.g. Norway, Ireland, New Zealand, UK), and many more are in the planning stages [10]. In 2012 the Australian Government announced the establishment of a national network of large offshore Commonwealth Marine Reserves (CMRs). The network comprises 59 CMRs and covers 3.1 million km^2^, making this the largest network of marine reserves in the world. A 10-year management plan is being developed for each reserve that identifies known biophysical characteristics of the reserve, sets out specific objectives that support the protection and conservation of biodiversity and other natural and cultural values, and provides for ecologically sustainable use of resources within the reserve (e.g. South-east CMR Network Management Plan 2013–2023).

For many parts of the reserve network of Australia, information to describe biological communities is lacking. This is particularly the case for the northern marine region where the CMR network design is based on the inferred significance of key ecological features (KEFs) in relation to biodiversity [11]. Among the KEFs identified for the northern region, the most extensive are the carbonate banks and terraces that cover approximately 72,000 km^2^ of the Sahul Shelf and Van Diemen Rise in the Timor Sea. These seabed features and the benthic communities they support are primarily incorporated within the Oceanic Shoals CMR in recognition that they are habitats thought to occur nowhere else in Australia [12]. While these hard ground habitats are known to support sponge and coral gardens [13], the biodiversity patterns of these communities remains poorly understood.

Sponges are receiving increasing attention in marine biodiversity research and associated management strategies due to the growing scientific and public awareness of their ecological and commercial importance [14]. Sponges are one of the major ecosystem engineers on the seafloor, providing habitat for a wide variety of species [15,16]. In addition, sponges play key ecological roles [17] in substrate modification [18], nutrient cycling [19], and microbial associations [20] and are being increasingly used in biodiversity and impact assessments [21,22]. Commercial applications for sponges include drugs via secondary metabolites to treat a range of microbial infections and cancers [23], anti-biofilm agents [24], and bath sponges [25]. Indeed, bioactive compounds that occur in several genera collected in the current study have been isolated for potential commercial applications [26–30].

Several environmental factors regulate sponge distributions, including wave exposure, light, temperature, sediment load, and substrate that directly affect the presence, abundance and morphology of sponges [31,32]. Depth, slope, and distance offshore can be proxies for these factors [31] and can also be related to sponge assemblage structure and abundance [33–35]. However, relationships between environmental factors and sponges are not always obvious or simple, as they can vary across sites or regions [34,36,37] or instead reflect a suite of variables that regulate biodiversity (e.g. depth, relief, substrate, and exposure in [36]). Due to the complexity of the potential relationships between this large range of potentially predictive variables, derived variables that incorporate multiple environmental variables (e.g. geomorphic features) may be more useful predictors than individual environmental variables. For example, topographically complex habitats supported significantly higher diversity than subdued geomorphic features along a 100 m depth gradient in the central Great Barrier Reef [38]. Similarly, a study of sponges in the eastern Oceanic Shoals CMR confirmed that raised geomorphic features (banks, ridges, terraces) had distinct assemblages, higher species richness, and more biomass of sponges than the surrounding plains and valleys [16].

This study is a follow-up to Przeslawski et al. [16] in which we add sponges from the western Oceanic Shoals CMR to the existing sponge species inventory from the eastern CMR; this broader regional approach allows a more management-focussed analysis of these sponge communities. We use a species-level inventory compiled from three marine surveys to investigate the patterns of sponge biodiversity on carbonate banks and other raised geomorphic features in a marine reserve in northern Australia. We address several questions relevant to marine management in the region at regional scales (i.e. entire reserve), meso-scales (i.e. eastern vs western areas of the reserve), and local scales (i.e. individual banks). These questions include: 1) Are carbonate banks and other raised geomorphic features associated with biodiversity hotspots? 2) Can environmental (depth, substrate hardness, slope) or biogeographic (east vs west) variables help explain local and regional differences in community structure? 3) Do sponge communities differ among individual raised geomorphic features? These patterns of sponge diversity are then considered in the context of marine reserve management in order to explore how such information may help support the future management of this region. For example, the study area’s proximity to existing gas fields and infrastructure means that marine environmental baselines should prove a valuable resource for industry environmental approval and compliance processes [39].

## Methods

### Surveys and Study Area

Biological and environmental data used in this study were collected on three surveys on the *R*.*V*. *Solander*: SOL4934 in 2009 and SOL5117 in 2010 to the eastern Oceanic Shoals CMR (1938 km^2^ mapped, sampled at 30–180 m depth) and SOL5650 to the western Oceanic Shoals CMR (507 km^2^ mapped, sampled at 36–90 m depth) (Fig 1). In addition, sponge samples from transit survey on the *R*.*V Southern Surveyor* in 2012 (SS2012t07) were opportunistically collected to provide a preliminary comparison between regions (Fig 1a, S1 Fig). The main study area was chosen to include part of the Oceanic Shoals Commonwealth Marine Reserve (CMR) and associated shallow carbonate banks and other raised geomorphic features (e.g. shoals, pinnacles in [40]).

Seven grids in the main study area were mapped using high-resolution multibeam (SIMRAD EM3002D, 300 kHz) (Fig 1). For the western region (survey SOL5650), sampling stations were chosen to target banks, as identified onboard from multibeam bathymetry and using a generalised random tessellation stratification (GRTS) design [41]. For the eastern region (surveys SOL4934, SOL5117), grids were chosen to represent geomorphic features typical of northern Australia such as banks, valleys, and plains; the results from sampling these other features have been previously published in Przeslawski et al. [16]. In the opportunistic study area, an additional two grids were mapped in 2005 (see [42] for details), while sponge samples were collected in 2011.

### Biological sampling

Epifauna were collected with an epibenthic sled towed for approximately 50–200 m at 1.5–2 knots (see S1 Table for transect coordinates). Samples were collected under EPBC reference numbers 2009/4951 (SOL4934) and 2010/5517 (SOL5117), in which proposed activities were not considered controlled actions, and permit number AU-COM2012-152 (SOL5650) (Department of Environment). Regression analyses confirmed that sled transect distance did not affect species richness (R^2^ < 0.001, p > 0.90) or biomass (R^2^ < 0.01, p > 0.30). The sled was 1.5 x 1 m (width x height) and fitted with a 6 m long, 45 mm stretch diamond net. Specimens were sorted to phylum and weighed. Each group was then separated into morphospecies to provide estimated taxonomic richness, and each morphospecies was photographed and preserved in ethanol for taxonomic identification. Subsamples of 100–1000 g from selected morphospecies (mainly sponge and cnidarians) were frozen for biodiscovery research focused on secondary metabolites. Taxonomic vouchers of sponges were deposited at the Museum and Art Gallery of the Northern Territory (MAGNT, formerly the Northern Territory Museum), and frozen samples were accessioned at the Australian Institute of Marine Sciences Bioresources Library.

### Species identifications

A thick section and spicule slide was prepared from each sponge voucher using standard methods [43,44], identified to genus following Hooper and Van Soest [45], and assigned to valid species and higher taxa as listed in the last consulted version of the World Porifera database [46] using available taxonomic literature. A unique code or operational taxonomic unit (OTU) was assigned to unknown or undescribed taxa, based on the museum registration number (e.g. *Scleritoderma* sp. NT0205). We used presence/absence data due to the colonial nature of many sponges and potential issues arising from sled sampling such as fragmentation and unstandardised effectiveness of collection [36].

### Environmental data

Bathymetric depth and backscatter values were acquired and processed from the multibeam sonar system as described in [47–49]. Backscatter measures seabed reflectance which can be used as a proxy for substrate hardness [50]. Slope was calculated using the Spatial Analyst toolbox in ArcGIS 10.0 on the bathymetric data. Averages and standard deviations of all environmental variables over each transect were determined using the ‘Zonal Statistics as Table’ tool in ArcGIS. The mean and standard deviation of slope were strongly correlated with each other (R^2^ = 0.75) which can exaggerate relationships; thus, only one of these factors (mean slope) was included in the current study [51]. See Table 1 for a description of environmental variables.

**Table 1:** Regression results of the relationships between environmental variables and sponge species richness. Environmental variables were square-root transformed to reduce skewness and heteroscedasticity.

a)	Factor	Description	R^2^	p
	Backscatter (mean)	Average substrate hardness of sled transect	0.000009	0.9780
	Bathymetry (mean)	Average depth of sled transect	0.0499	0.0979
	Backscatter (stdev)	Variation in substrate hardness of sled transect	0.000003	0.9870
	Bathymetry (stdev)	Variation in depth of sled transect	0.0007	0.8108
	Slope (mean)	Average slope of sled transect	0.0016	0.7210

### Statistical analyses

Diversity was estimated using sponge species richness and species assemblage structure (presence/absence species matrix). To assess the relationships between sponges on raised geomorphic features and environmental factors (see Table 1), regression analyses were used on richness data, and a distance-based linear model (DistLM) using stepwise selection was applied to assemblage data. The DistLM procedure fits environmental variables to a Bray-Curtis similarity matrix (including presence/absence) to quantitatively estimate their relationship to sampled biological variation [52]. Environmental variables were square-root transformed to reduce skewness and heteroscedasticity [52]. To test for differences between the species richness estimates of the sponge communities on raised geomorphic features between and within our eastern and western study areas, 1-factor Analyses of Variance (ANOVAs) were used. Non-metric multidimensional scaling (n-MDS) plots and analyses of similarities (ANOSIMs) were used to assess differences in assemblage structure, with both full assemblages and assemblages excluding singletons (i.e. species collected only once within a given dataset). All multivariate analyses were performed using PRIMER (v.6.1.14), and all univariate analyses with the R statistical platform (v 3.3.1). Unless otherwise stated, all analyses were performed only on data collected from the main study area.

## Results

### Summary of collections

**Fig 2:**
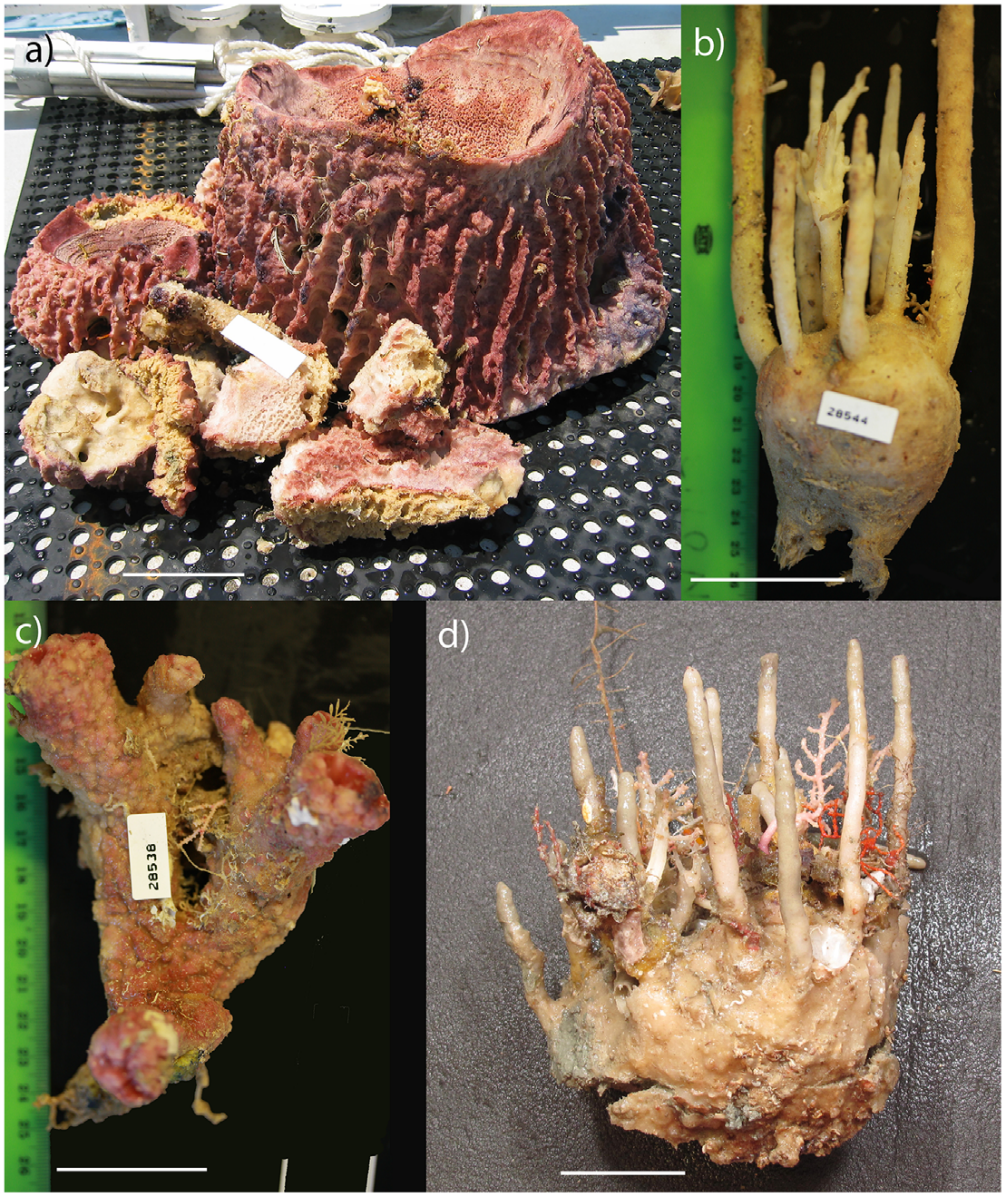
Specimen images of the most common sponge species collected from the Oceanic Shoals CMR: a) *Xestospongia testudinaria* collected from SOL4934 at 28 m, b) *Oceanapia* sp. NT0185 collected from 82 m, c) *Scleritoderma* sp. NT0205 collected from 82 m, and d) *Oceanapia* sp. NT0186 with associated epifauna collected from 63 m. All depths specified are means of the sled transect. Scale bar is 5 cm.

In the main study area, sleds were deployed at 106 sites (56 sites on raised geomorphic features), with sponges collected from 86 of these (45 sites on raised geomorphic features) (Fig 1) (S1 Table). Standardised species richness ranged from 0–85 species per 100 m sled tow (Table 2). Approximately 750 sponge specimens were collected and assigned to 348 species, representing four classes, 55 families and 133 genera (Table 3, S2 Table). Of the 348 species, 91% were collected on raised geomorphic features. The majority of species (337) collected belong to the class Demospongiae, four species to Calcarea, six species to Homoscleromorpha, and one species to Hexactinellida (Table 3). Only 18% of all species were associated with taxonomically described and named species. The remaining species were assigned to unique OTUs (S2 Table) but remain either undescribed or require additional taxonomic work and comparison with type specimens to be further determined. The most common species on banks and other raised features in the main Oceanic Shoals CMR study area were *Xestospongia testudinaria* (collected at 32 stations), *Scleritoderma* sp. NT0205 (21 stations), *Oceanapia* sp. NT0186 (18 stations), and *Oceanapia* sp. NT0185 (18 stations) (Fig 2).

**Table 2:** List of standardised numbers of sponges species collected from each sled transect.

Species richness				Species richness			
Station	Sled distance	Absolute	Standardised^a^	Station	Sleddistance	Absolute	Standardised^a^
**SOL4934(east)**				**SOL5650(west)**			
001BS021	122	8	6.6	006BS001	133	1	0.8
002BS019	138	10	7.2	014BS002	133	8	6.0
003BS027	394	6	1.5	015BS003	178	14	7.9
005BS002	180	18	10.0	016BS004	122	36	29.5
006BS003	84	0	0.0	024BS005	131	4	3.1
009BS006	70	0	0.0	025BS006	109	6	5.5
014BS011	59	0	0.0	026BS007	133	20	15.0
021BS012	212	19	9.0	029BS008	132	14	10.6
022BS013	110	26	23.6	031BS009	122	2	1.6
023BS014	109	33	30.3	036BS010	146	9	6.2
025BS015	166	33	19.9	037BS011	142	17	12.0
029BS017	135	0	0.0	038BS012	44	0	0.0
031BS018	112	10	8.9	044BS013	31	0	0.0
032BS020	174	23	13.2	046BS014	34	22	64.7
033BS022	128	3	2.3	047BS015	25	0	0.0
034BS023	138	16	11.6	053BS016	54	9	16.7
041BS028	133	25	18.8	055BS017	35	18	51.4
063BS045	232	1	0.4	056BS018	40	33	82.5
**SOL5117(east)**				061BS019	122	22	18.0
023BS018	204	7	3.4	063BS020	35	12	34.3
024BS019	306	2	0.7	073BS021	47	14	29.8
025BS020	140	6	4.3	074BS022	46	39	84.8
028BS022	155	8	5.2				
035BS023	67	14	20.9				
044BS026	120	32	26.7				
045BS027	55	13	23.6				
050BS030	141	4	2.8				
051BS031	187	6	3.2				
052BS032	159	17	10.7				
055BS034	366	23	6.3				
064BS035	171	19	11.1				
065BS036	135	27	20.0				
073BS038	111	2	1.8				
081BS040	133	12	9.0				
082BS041	122	23	18.9				

^a^ per 100 m tow of 1.5 x 1 m sled.

**Table 3:** List of taxa recorded from all study and opportunistic areas of this study. Numbers in parenthesis indicate the number of OTUs recorded per taxon if it was greater than one. A question mark represents uncertainty associated with that identification. Class is shown by underlined capital text; order by capital text, family by bold text, and genus and species by italics. A full list including OTUs codes and specimen numbers per taxon can be found in S2 Table.

CALCAREA	
CLATHRINIDA	**Clathrinidae:** *Clathrina*. **Leucetiidae:** *Leucetta*. **Soleneiscidae (2):**
DEMOSPONGIAE	
Agelasida	**Agelasidae:** *Agelas* (5)
Astrophorida	**Ancorinidae:** *Asteropus*, *Disyringa dissimilis*, *Ecionemia* (2), *Holoxea*?, *Jaspis (2)*, *Melophlus sarasinorum*, *New genus A*?, *Psammastra*, *Rhabdastrella globostellata*, *Stelletta (4)*, *Stelletta clavosa cf*, *Stryphnus*. **Calthropellidae:** *Chelotropella*, *Chelotropella*? (2), *Pachastrissa*. **Geodiidae:** *Erylus*, *Geodia* (7). **Pachastrellidae:** *Poecillastra*?
Chondrosida	**Chondrillidae:** *Chondrilla australiensis*, *Chondrosia*? (2)
Dendroceratida	**Darwinellidae:** *Chelonaplysilla*, *Darwinella*, *Dendrilla mertoni*. **Dictyodendrillidae:** *Dictyodendrilla* (2)
Dictyoceratida	**Dysideidae:** *Dysidea* (6). **Irciniidae:** *Ircinia* (2), *Ircinia irregularis*, *I*. *pinna*, *Psammocinia* (2), *Sarcotragus*. **Spongiidae:** *Coscinoderma* (2), *Euryspongia*?, *Hyattella* (3), *H*. *intestinalis*, *Leiosella* (4), *Rhopaloeides*, *Spongia* (4). **Thorectidae (6):** *Aplysinopsis* (3), *Cacospongia*, *Dactylospongia*?, *Fascaplysinopsis*, *Fasciospongia*, *Hyrtios* (2), *Luffariella variabilis*, *Petrosaspongia*?, *Phyllospongia*, *Semitaspongia*, *Thorecta*, *Thorectandra excavatus*, *Thorectandra*? (2)
Hadromerida	**Clionaidae:** *Cliona* (3), *Spheciospongia congenera*, *S*.*vagabunda*. **Polymastiidae:** *Polymastia*?. **Suberitidae:** *Aaptos* (2). **Tethyidae:** *Tethya* (4), *Tethytimea*
Halichondrida	**Axinellidae:** *Axinella aruensis*, *Cymbastela vespertina*, *Phakellia tropicalis*, *Phakellia*, *Phycopsis*, *Reniochalina*, *Reniochalina stalagmitis*. **Bubaridae:** *Bubaris*?. **Dictyonellidae:** *Acanthella* (3), *Acanthella cavernosa*, *Acanthella pulcherrima*, *Phakettia euctimena*, *Scopalina*?, *Stylissa carteri*. **Halichondriidae:** *Amorphinopsis excavans*, *A*. *fenestrata*, *Amorphinopsis*, *Axinyssa* (4),*Ciocalypta stalagmites*, *Epipolasis*, *E*. *suluensis*, *Halichondria*, *H*. *carotenoidea*, *H*. *phakellioides*, *H*. *stalagmites*, *H*. *carotenoidea*, *Halichondria* (2), *Spongosorites*?, *Topsentia* (2). **Heteroxyidae:** *Higginsia*, *Higginsia mixta*, *H*. *strigilata cf*., *Myrmekioderma*, *M*. *Granulatum*, *Parahigginsia*
Haplosclerida	**Callyspongiidae:** *Callyspongia (5)*, *C*. *biru*. **Chalinidae:** *Haliclona* (3), *H*. *(Gellius)*, *H*. *(Haliclona (Halichoclona)* (2), *H (Haliclona)* (2), *H*. *(Reniera)*. **Niphatidae:** *Amphimedon* (3), *Cribrochalina*, *Gelliodes* (4), *G*. *fibulata*, *Hemigellius*(3), *Niphates* (5). **Petrosiidae:** *Neopetrosia* (2), *N*. *exigua*, *Petrosia* (8), *P*. *alfiani*?, *P*. *durissima*?, *P*. *(P*.*) lignosa*, *P*. *(Strongylophora)*, *Xestospongia* (10), *X*. *testudinaria*. **Phloeodictyidae:** *Oceanapia* (8)
Haplosclerida + Dictyoceratida^A^	**Niphatidae + Thorectidae:** *Gelliodes + Dysidea*
Lithistida (2)	**Azoricidae:** *Leiodermatium* (4). **Corallistidae:** *Neophrissospongia*?. **Desmanthidae:** *Petromica*. **Scleritodermidae:** *Aciculites*, *Microscleroderma* (2), *Scleritoderma* (2). **Siphonidiidae:** *Gastrophanella*. **Theonellidae:** *Manihinea* (2), *Racodiscula* (2), *Theonella* (12). **Vetulinidae:** *Vetulina*?
Poecilosclerida:	**Acarnidae:** *Acarnus*, *Cornulum*, *Damira*, *Chondropsis*. **Coelosphaeridae:** *Coelosphaera* (2), *Lissodendoryx* (2). **Crellidae:** *Crella* (3). **Desmacellidae:** *Biemna*, *B saucia*. ***Hymedesmiidae*?, Iothrochotidae:** *Iotrochota*, *I*. *baculifera*. **Isodictyidae:** *Coelocarteria agglomerans*, *C*. *singaporensis*. **Microcionidae:** *Antho (Plocamia)*. **Microcionidae:** *Clathria* (3), *C*: *abietina*, *C*. *cervicornis*, *C*. *ramosa*, *C*. *(Thalysias)* (2), *C*. *(T*.*) abietina*, *C*. *(T*.*) erecta*, *C*. *(T*.*) ramosa*, *C*. *(T*.*) vulpina*. ***Microcionidae*?**. **Mycalidae:** *Mycale* (3). **Myxillidae:** *Myxilla*? (2), *Psammochela*, *P*.*fibrosa*, *P*. *tutiae*, **Raspailiidae:** *Amphinomia*, *A*. *sulphurea*, *Axechina raspailioides*, *Ceratopsion* (5), *C*. *axifera*, *C*. *palmatum*, *Echinodictyum* (2), *E*. *asperum*, *E*. *cancellatum*, *E*. *mesenterinum*, *Ectyoplasia* (2), *E*. *tabula*, *E*.*vannus*, *Endectyon* (2), *Eurypon*, *Raspailia*, *R*. *phakellopsis*, *R*. *vestigifera*, *Thrinacophora cervicornis*, *Trikentrion flabeliforme*. **Tedaniidae:** *Tedania*
Spirophorida	**Tetillidae:** *Cinachyrella* (4), *C*. *australiensis*, *Paratetilla*, *Tetilla* (2)
Verongida	**Aplysinellidae:** *Aplysinella*, *Suberea* (3). **Ianthellidae:** *Ianthella* (2), *I*. *basta*, *I*. *flabelliformis*, *I*. *reticulata*? **Pseudoceratinidae:** *Pseudoceratina* (4)
HEXACTINELLIDA	
	Lyssacinosida
HOMOSCLEROMORPHA:	
	Homosclerophorida: **Plakinidae:** *Plakinastrella* (2), *Plakortis* (4)

^A^ These two species were always found together, one growing upon the other, and almost certainly represent a mutualism or commensalism.

In the opportunistic study area, sleds were deployed at ten sites, with sponges collected from all but one of these. A total of 55 species were collected from eight sites (S1 Fig), including 22 species that were not collected from the main Oceanic Shoals CMR study area (S2 Table). From the entire collection (main and opportunistic study areas), most species (60%) were collected only once (i.e. collected at one station) or twice (12%) (Supplementary Material C).

**Fig 3:**
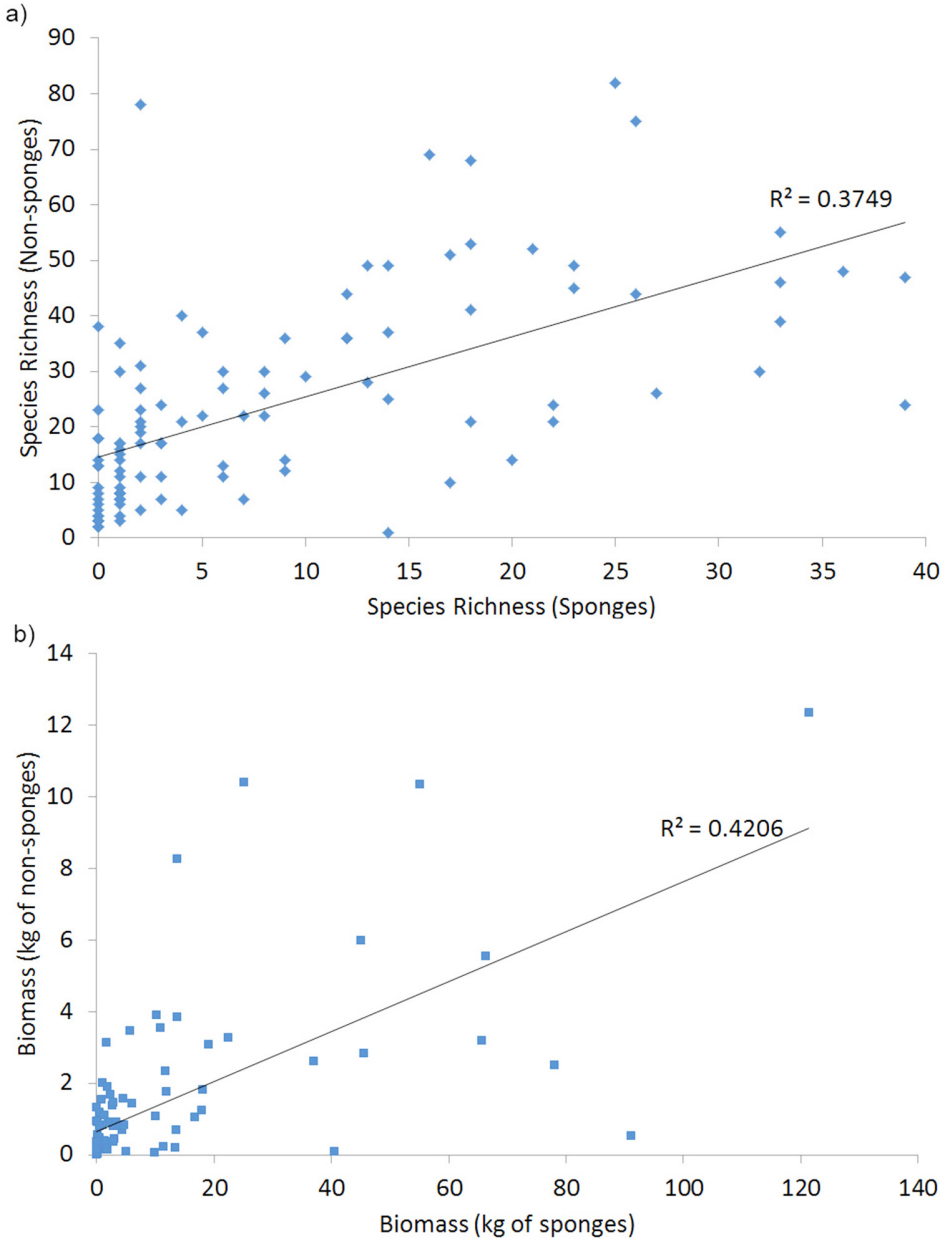
Relationship between sponges and other taxa based on a) richness and b) biomass, the latter excluding outliers due to weight of hard corals collected at station 16BS04 from SOL5650 (23.4 kg hard corals) and station 55BS35 from SOL5117 (100.9 kg hard corals).

Based on onboard estimates of taxonomic richness of non-sponge organisms, sponges are positively and significantly related to other taxa in respect to richness (R^2^ = 0.37, p < 0.0001) (Fig 3a) and biomass (R^2^ = 0.42, p < 0.0001) (Fig 3b), the latter after the exclusion of outliers at station 55 (SOL5117) and station 16 (SOL5650) due to large numbers of scleractinian corals and associated non-living material in the catch.

### Biogeographic and environmental relationships

**Fig 4:**
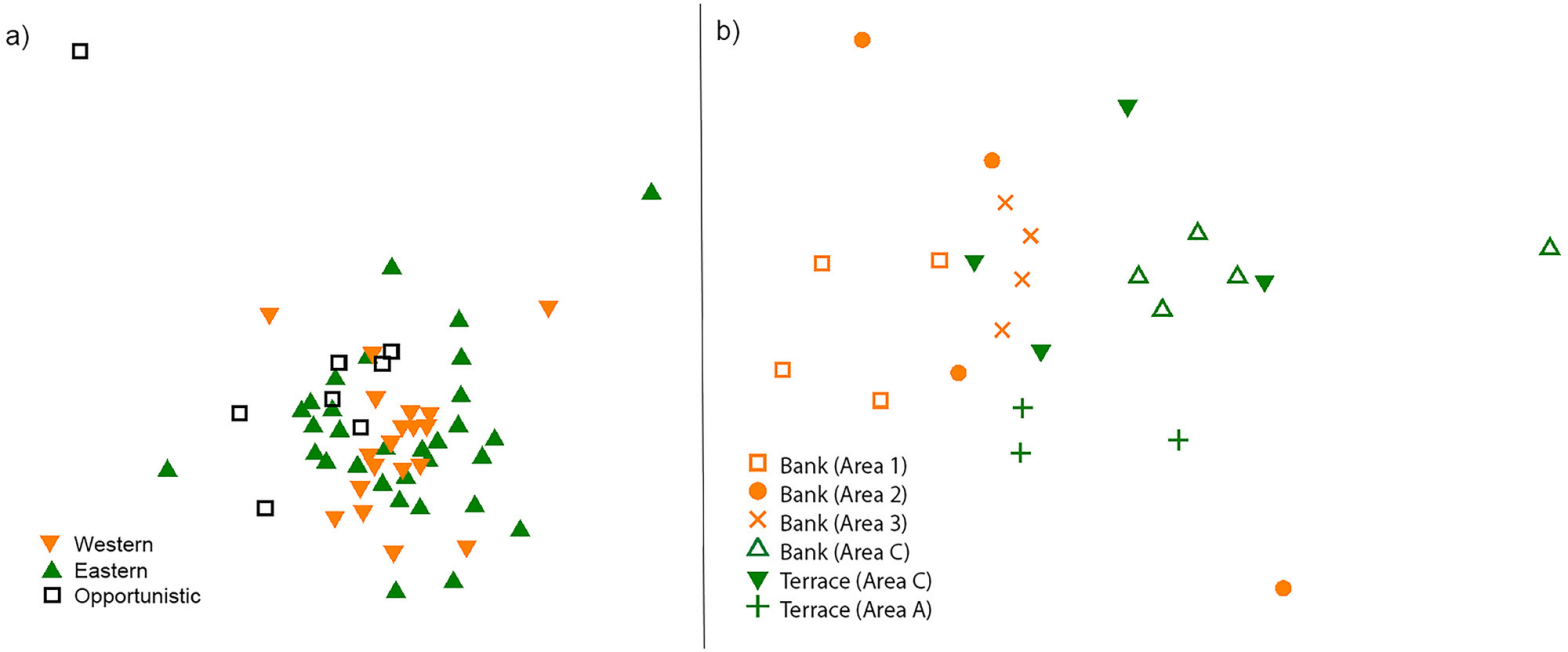
Non metric multidimensional scaling (n-MDS) plots using Bray-Curtis similarities for sponge assemblages in which singletons were excluded, showing collected from a) all raised geomorphic features, excluding an outlier at Station 63 from SOL4934 at which only one non-singleton species was collected (stress = 0.16) and b) raised geomorphic features at which sponges were collected from three or more sites in the western CMR (orange, numeric codes) and eastern CMR (green, alphabetic codes) stress = (0.014). Locations of each geomorphic feature are shown in Fig 1. Each point on an MDS represents an assemblage collected at a site, and increasing distance between points indicates decreasing similarity.

There was no difference between sponge species richness on raised geomorphic features in the eastern, western, and the opportunistically sampled study areas (ANOVA: df = 2, F = 0.316, p = 0.730). Similarly, species assemblages on raised geomorphic features were not significantly different between any of the study regions (ANOSIM: Global R = 0.036, p = 0.203). However, when singletons were excluded from the analysis, results show marginally significant differences in assemblages between the western and opportunistic study areas (ANOSIM: Global R = 0.091, p = 0.033) (Fig 4a).

On raised geomorphic features in the study area, sponge species richness was not significantly linearly related to any environmental factor (Table 1). However, sponge assemblages on raised geomorphic features were significantly related to mean backscatter, mean bathymetry, and slope (Table 4a). These relationships were not strong, as shown by the best modelled representation of the data using AIC in which mean backscatter and bathymetry explained only 9% of assemblage variation in the total dataset (Table 4a). When eastern and western regions were analysed separately there were no strong relationships evident, with the best predictive model for each region only containing a single factor that explained no more than 10% of assemblage variation (western region: mean bathymetry, R^2^ = 0.106; eastern region, mean backscatter, R^2^ = 0.0780) (Table 4b and 4c). Relationships did not change when singletons were excluded from the assemblage dataset.

**Table 4:** Results from the marginal and sequential tests of distance-based linear (DistLM) models on sponge assemblages with a) the full dataset, b) the western dataset, and c) the eastern dataset. Environmental variables were square-root transformed to reduce skewness and heteroscedasticity, and a stepwise selection was used for sequential tests.

	Marginal tests			Sequential tests		
	Factor	Pseudo F	p	Factor	AIC	R^2^
a)	Bkstr (mean)	2.5235	0.001	+Bkstr (mean)	408.66	
	Bathy (mean)	2.4089	0.001	+Bathy (mean)	408.34	0.0949
	Bkstr (stdev)	1.5021	0.018			
	Bathy (stdev)	1.3804	0.051			
	Slope (mean)	1.9037	0.002			
b)	Bkstr (mean)	1.6904	0.022	+Bathy (mean)	148.65	0.106
	Bathy (mean)	1.8971	0.003			
	Bkstr (stdev)	1.3186	0.094			
	Bathy (stdev)	1.4433	0.057			
	Slope (mean)	1.5103	0.036			
c)	Bkstr (mean)	2.4517	0.001	+Bkstr (mean)	259.61	0.0780
	Bathy (mean)	2.4358	0.001			
	Bkstr (stdev)	1.2024	0.152			
	Bathy (stdev)	1.5107	0.025			
	Slope (mean)	1.0503	0.353			

### Biodiversity patterns between individual geomorphic features

At least three sleds successfully collected sponges at each of six raised geomorphic features (three in the west, three in the east) (Fig 1b and 1c), thus allowing comparisons among these features of richness and assemblages patterns. Sponge species richness was not significantly different among these raised geomorphic features (ANOVA: df = 5, F = 0.535, p = 0.7474), but sponge assemblages were (ANOSIM: Global R = 0.328, p < 0.001) (Fig 4b). Pairwise comparisons from the ANOSIMs confirmed that there were significant differences between assemblages on an eastern bank (Bank C) and the western banks (Banks 1, 2, 3) (R = 0.356 to 0.681, p = 0.008 to 0.024). Assemblages on the eastern terraces (Terraces A, C) were significantly different from assemblages on the western Banks 1 and 3 (R = 0.323 to 0.639, p = 0.029 to 0.057), although they were not significantly different to those from Bank 2, indicating that east-west differences may operate at the scale of individual geomorphic features. There were also significant differences in assemblages between Banks 1 and 3 in the west (R = 0.542, p = 0.029). Similar patterns occurred when singletons were excluded, with the exception of significant differences between Banks 2 and 3 in the west (R = 0.302, p = 0.029). Within each raised geomorphic feature, assemblages ranged from relatively similar (e.g. Bank 3: Bray Curtis similarities 32.258–40.909) to different (e.g. Bank 2 in the west: Bray Curtis similarities 0–29.412), as indicated by the clustering patterns of points in an n-MDS (Fig 4b).

## Discussion

### Biodiversity hotspots

Biodiversity hotspots have been linked to production and exportation of species [53] and the maintenance of global marine biodiversity [54]. Identifying and defining biodiversity hotspots is challenging [55] and often varies according to target taxa, spatial scale, or metric (e.g. richness, endemism, diversity index) [56,57]. We adopt the definition of Hooper and Ekins [58] to define a sponge biodiversity hotspot as >250 species found within a given bioregion. According to this definition, the banks and other raised features of the Oceanic Shoals CMR constitute a sponge biodiversity hotspot. Sponge biodiversity hotspots have been identified elsewhere in northern Australia, including the northern Wessel Islands and Darwin/Coburg region [58,59], but spatial data gaps still hamper broad-scale assessment of many areas (Fig 1a). For example, a synthesis of Australian marine faunal databases in 2010 identified 17 higher taxonomic groups from which species numbers may be estimated for each Large Marine Domain (LMD) [7]. The current study overlaps the Northern LMD which spans the western Oceanic Shoals CMR to Cape York, and the estimated number of described and undescribed sponge species in this large area was 125 [7]. The current study yielded 370 sponge species from four surveys spanning only a very small portion of the Northern LMD (Fig 1a), thus indicating that biodiversity of sponges (and possibly other taxa) may be much higher than previously thought.

Sponge biodiversity hotspots can indicate high levels of biodiversity in other taxa at a given location [16], as also supported by the current study. Many species of sponge collected in the current study are habitat providers (Fig 2) which explains the correlation between sponge species richness and that of other taxa. Previous studies have also confirmed the importance of sponges in providing structural complexity and habitat [15,16,20,60]. As such, sponges are one of the key surrogate taxa for biodiversity assessments in northern Australia and may be an appropriate group to target for monitoring activities.

### Biogeographic and environmental relationships

Sponge biodiversity (as measured by richness and assemblages) was not significantly different between the eastern and western CMR, although there were potential differences in assemblages between the western Oceanic Shoals CMR and the Wessel Island CMR region over 500 km away. This indicates that at the regional scale, the Oceanic Shoals CMR contains similar sponge communities and richness levels throughout the reserve, thereby supporting the management of the CMR as a single large reserve. However, such broadscale regional analyses do not account for changes in species assemblages at finer spatial scales [61] and therefore do not negate the need for monitoring at both the east and west sides of the CMR, as well as along known environmental gradients [62] or among individual geomorphic features. This is shown in the current study by east-west differences among pairs of geomorphic features (e.g. Bank C in east and Banks 1, 2, and 3 in west) but not others (Terrace C in east and Bank 2 in west). Additionally, environmental drivers of sponges may differ between the eastern and western banks of the CMR; our results revealed that bathymetery had the strongest relationship to sponge assemblages in the west, while backscatter had the strongest relationship to assemblages in the east.

There is a growing body of research investigating biodiversity patterns within and between marine reserves [63], but most of these focus on comparatively small reserves (e.g. <2500 hectare reserves in [64] compared to 7,174,400 hectares of the Oceanic Shoals CMR). There are few studies of biodiversity patterns at large reserve scales, and these suggest differences both within and between reserves, variations of which seem dependent on environmental gradients and heterogeneity encompassed within a given area. For example, reserves in the tropical eastern Pacific showed obvious differences in fish biomass and macroinvertebrate abundances between eastern and western study areas, reflecting environmental differences associated with oceanic and continental reserves [65]. In contrast, the eastern and western study areas in the Oceanic Shoals CMR are approximately the same distance along the continental shelf. In some taxa, dispersal potential may regulate broad-scale spatial differences (e.g. algae in [63]), although neither oviparous nor viviparous sponge assemblages showed any relationship to distance between sites [66].

Sponge species richness and community structure on raised geomorphic features were not associated with any strong and significant linear environmental gradients. It may simply be that sponge assemblages from the Van Diemen Rise are regulated by other factors not considered here (e.g. ocean currents, light availability, sedimentation) [31]. Alternatively, a coarser biological metric such as community type (e.g. sponge-dominated vs octocoral-dominated as per [39]) may be more strongly related to environmental factors analysed here. It may also be that the range of environmental factors analysed here was not large enough to elicit a response; for example, our depth range was 22–96 m, with previous studies finding stronger relationships between depth and sponges at different (e.g. < 50 m in [67]) or broader (e.g. 48–195 m in [68]) depth ranges. Although our study revealed no clear abiotic surrogate for sponge biodiversity, the identification of environmental relationships is still useful to investigate ecosystem processes and inform further research and management plans. In the current study, mean depth and substrate hardness explained more variation in sponge assemblages than other factors examined (Table 4a), with depth a stronger driver in the west (Table 4b) and substrate hardness a stronger driver in the east (Table 4c). These variables are encompassed in geomorphic features which were found to affect sponge assemblages in Przeslawski et al [16]. These findings support current knowledge of sponge ecology, with depth and substrate hardness previously associated with sponge assemblages and abundance [35,36,69]. Thus, areas of hard ground could be identified to manage sponge gardens and associated high biodiversity, and a range of depths should be included to appropriately represent distinct sponge assemblages.

The utility of abiotic surrogates depends on the spatial scale of interest [31], and changing sampling scales also changes our ability to detect ecosystem processes [70]. For example, in the current study comparing assemblages over scales of tens of kilometres, environmental relationships between sponge biodiversity and depth, substrate hardness and slope were non-existent or weak. However, at a finer spatial scale, surface topography and substrate complexity can affect biodiversity by regulating the larval settlement of sponges and corals [71]. Indeed, sponge assemblages in Indonesia were significantly related to remotely sensed variables such as offshore distance, coral formation area, and exposure but were unrelated to distance between sites [66]. If marine management plans were to specify the spatial scale at which a given strategy or action is operating, appropriate environmental gradients or surrogates for biodiversity could be identified and sampled.

### Biodiversity patterns between individual geomorphic features

Sponge assemblages can vary among individual banks and other raised geomorphic features in the Oceanic Shoals CMR, but this pattern is not universal among all banks, suggesting that more research is needed to determine environmental or biological regulators of sponge community differences among banks. The proximity of raised features to one another may affect the similarity of sponge assemblages, as possibly shown by the similarities between sponge assemblages on relatively close Terrace C and Bank C and the differences between relatively distant eastern and western raised geomorphic features (Fig 4). Our results are inconclusive, however, due to the low numbers of banks analysed, and further research incorporating more banks at various distances from each other is needed. Other research has shown that sponge assemblages are unrelated to distances between reef sites [66], although the distance between the furthest reef sites was less than that of the current study (~100 km vs 300 km). Nevertheless, we show that at least some assemblages are significantly different among banks, and marine managers may thus consider a management plan for the Oceanic Shoals CMR that accounts for these smaller spatial scales (i.e. 1–2 kilometers) while also balancing the practicality of enforcement issues at such fine scales. This does not contradict our regional analysis in which no difference in sponge assemblages between east and west were detected. Rather, our results highlight the importance of spatial scale in biodiversity assessments and associated management strategies. Importantly, assemblage similarity among banks is not only associated with environmental and ecological characteristics of a particular bank, but also with the dispersal potential and connectivity of individual species [72]. This is discussed further in the next section.

### Management implications of findings

After the establishment of marine reserves, the focus of management authorities typically shifts from discovery and description to monitoring. This shift helps to gauge the effectiveness of specific management actions (e.g. exclusion of bottom trawling) and also to identify potential impacts from human use. However, the need for some degree of ongoing discovery and description remains as shown by the number of new species collected on marine surveys to new areas [3,73]. This underscores the fact that baseline data is still lacking [4,74], particularly for northern Australia [7,16] and associated sponges (Fig 1a).

Even with species inventories, conservation planning requires spatially-explicit data on both the environment of the region of interest and the taxa of conservation concern [75]. Such data are invariably incomplete, particularly in the marine realm [2,76]. Conservation planning exercises therefore often rely on environmental or taxonomic surrogates to represent general patterns in biodiversity [77,78], but such surrogates are often limited in their utility among regions, ecosystems, metrics, and taxonomic resolutions [79–81]. Species distribution models are powerful and commonly used tools for identifying priority areas for conservation actions [82,83]. However, the accuracy of species distribution models, and therefore the success of conservation actions, can be undermined by imprecise species occurrence records [84]. Systematic collection of both biological specimens and a range of environmental data are therefore necessary to provide managers with the necessary tools to make informed decisions regarding the location of priority areas for conservation. For example, protection of regions with high sponge richness in the Oceanic Shoals CMR (e.g. raised geomorphic features in [16]) from potential threats such as bottom trawling would likely yield significant benefits for a range of other taxa, given the important role of sponges as habitat-forming taxa in the region. In addition, the congruence between areas of high species richness of sponges and other taxa suggests that sponges may provide excellent taxonomic surrogates for conservation planning in the Oceanic Shoals CMR [16] but see [79–81]. Sponge communities can also be an indicator of an important ecosystem service because they indicate the presence of hard ground, a limited resource in the marine environment which supports distinct communities potentially vulnerable to disturbance [85]. The ability of both taxonomic and environmental surrogates to reflect general biodiversity patterns is variable, depending on factors such as spatial scale, analytical method, or the ecosystem in question [31,79,86]. The spatial distributions of sponges reported here may provide valuable information for implementing robust conservation actions in a remote and poorly studied but diverse marine region.

The results of baseline inventories, such as the study presented here, are also essential inputs into regional-scale models of biogeographic processes and for refining and testing such models. For example, a regional-scale model of connectivity has been developed for northern Australia using ophiuroids as a model species [87]. Ophiuroids are generally considered to be passive drifters [88], and sponges exhibit similar behavioural characteristics [89], although they spend considerably less time in the water column on average [minutes to days for sponges, versus weeks for echinoderms; [90,91]. The significant differences in assemblages among individual banks and terraces highlight the importance of local geomorphic features in shaping community structure, which is consistent with the short pelagic larval durations associated with sponge larvae as well as asexual reproduction through fragmentation [92]. At a larger spatial scale, the lack of differences observed between the eastern and western Oceanic Shoals CMR suggests an extensive mix of frequent, but highly stochastic connections when taken at a larger regional scale. Population genetic analysis of the sponge specimens collected in the current study would help to quantify the direction and magnitude of exchange which could be directly compared with the connectivity model results. There are only a few taxa with sufficient sample size for this work (e.g. *Xestospongia testudinaria*), but due to wide distribution these may be less likely than uncommon species to show patterns, although research is still warranted. Genetic analysis could also be conducted with other specimens collected during the surveys (e.g. polychaetes in [39] to identify differences in distribution patterns between taxa (Annelida vs Porifera), habitats (epifaunal vs infaunal), or developmental mode (lecithotrophic vs planktotrophic). Developing an improved understanding of biophysical processes and connectivity can help inform management actions by identifying natural biogeographic regions [93,94], by detecting critical pathways of exchange [95], or by assessing the potential for transboundary exchange of resources or environmental risk [96].

The current study has used species-level identifications of an ecologically important group to address several questions related to marine management in a large tropical marine reserve. Importantly, we have further increased the evidence that the carbonate banks and other raised geomorphic features of the Van Diemen Rise and Sahul Shelf indeed constitute key ecological features that similarly operate at locations in the eastern and western part of the Oceanic Shoals CMR at a broad spatial scale. The associated species inventory will provide a foundation from which future predictive habitat, biodiversity and connectivity maps may be generated. Results may also be integrated with socioeconomic considerations [97] and inform future marine management plans, particularly as they relate to multiple spatial scales.

## Supporting Information

S1 Fig Map of study area for survey SS2012t07, with sled transects from that survey overlaid on bathymetry previously collected from [42]. (JPG)

S1 Table List of stations at which benthic sleds were deployed. (DOC)

S2 Table List of all sponge species collected from surveys SOL4934, SOL5117, SOL5650 and SS2012t07. (XLSM)
