# Supplementary figures and images for: Implications of Sponge Biodiversity Patterns for the Management of a Marine Reserve in Northern Australia
Source: PLoS One. 2015 Nov 25;10(11):e0141813. doi: 10.1371/journal.pone.0141813 (PMC4659674; doi:10.1371/journal.pone.0141813)

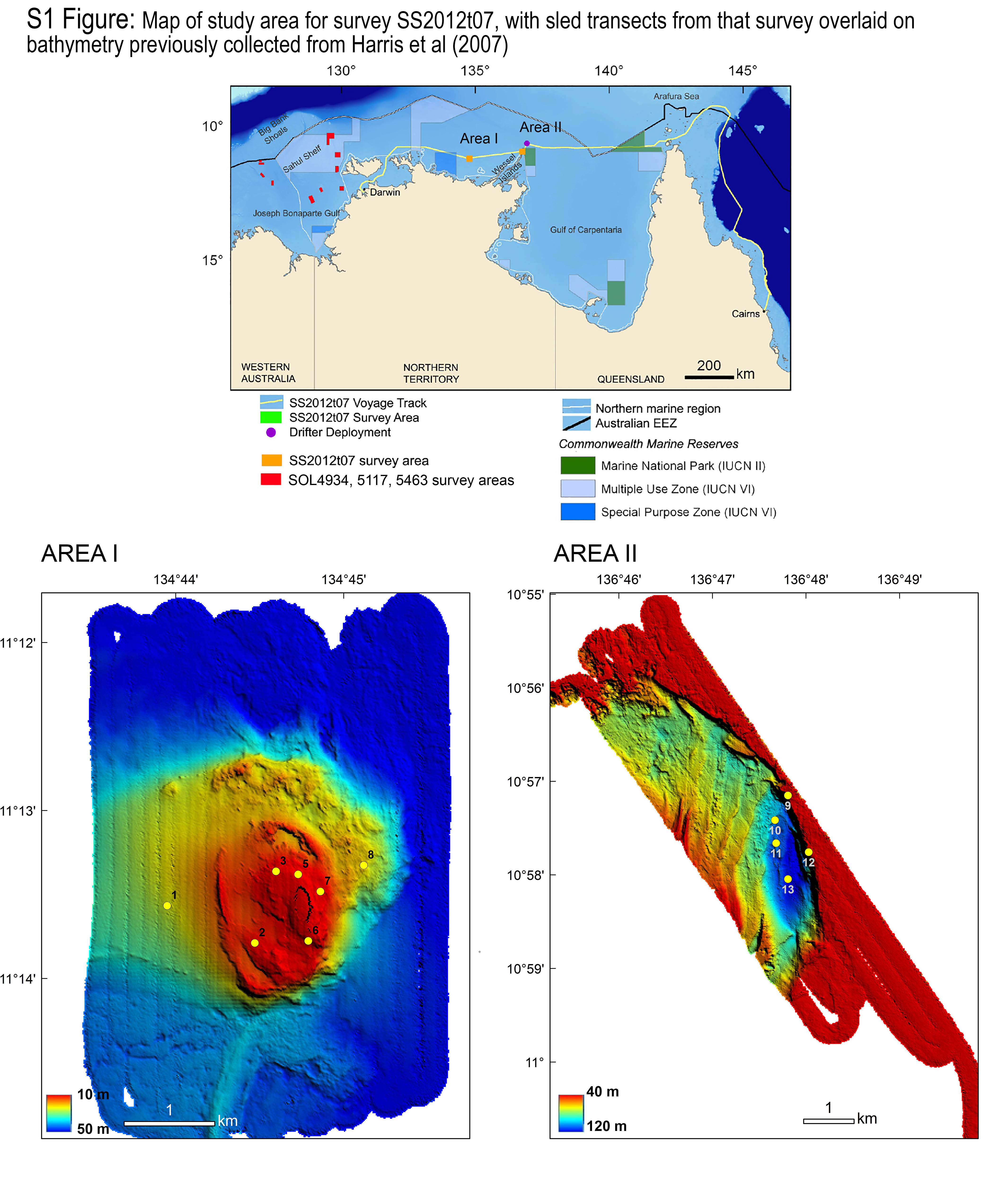

Supplement: S1 Fig — (JPG) [file pone.0141813.s001.jpg]
