# Supplementary material for: Implications of Sponge Biodiversity Patterns for the Management of a Marine Reserve in Northern Australia
Source: PLoS One. 2015 Nov 25;10(11):e0141813. doi: 10.1371/journal.pone.0141813 (PMC4659674; doi:10.1371/journal.pone.0141813)
Supplement: S1 Table — (DOC) [file pone.0141813.s002.doc]

Przeslawski R^1^, Alvarez B, Kool J, Bridge T, Caley MJ, Nichol S. Implications of sponge biodiversity patterns for the management of a marine reserve in northern Australia

^1^ Geoscience Australia, [rachel.przeslawski@ga.gov.au](mailto:rachel.przeslawski@ga.gov.au)

*S1 Table List of sites at which benthic sleds were successfully deployed. NB: Banks, terraces, and ridges are considered ‘raised geomorphic features’ for the purposes of our study.*

| **Site** | **Geomorph Feature** | **Study Area** | **Sponges Collected?** | **Start Lat** | **Start Long** | **End Lat** | **End Long** |
| --- | --- | --- | --- | --- | --- | --- | --- |
| 6A | Bank | East (A) | No | -10.375 | 129.7134 | -10.3756 | 129.7132 |
| 7A | Bank | East (A) | No | -10.3076 | 129.6804 | -10.3081 | 129.6802 |
| 8A | Bank | East (A) | No | -10.3753 | 129.646 | -10.3768 | 129.6448 |
| 50B | Bank | East (A) | Yes | -10.6802 | 129.5095 | -10.6805 | 129.509 |
| 51B | Bank | East (A) | Yes | -10.641 | 129.5275 | -10.6414 | 129.5273 |
| 52B | Bank | East (A) | Yes | -10.6296 | 129.4735 | -10.6297 | 129.4739 |
| 55B | Bank | East (A) | Yes | -10.6056 | 129.4832 | -10.6055 | 129.4837 |
| 9A | Plain | East (A) | No | -10.3947 | 129.6067 | -10.3955 | 129.6061 |
| 5A | Ridge | East (A) | Yes | -10.4669 | 129.7007 | -10.4662 | 129.7004 |
| 14A | Ridge | East (A) | Yes | -10.3058 | 129.6169 | -10.3071 | 129.616 |
| 23B | Ridge | East (A) | Yes | -10.4469 | 129.6698 | -10.4464 | 129.6697 |
| 24B | Ridge | East (A) | Yes | -10.4481 | 129.7174 | -10.4485 | 129.7175 |
| 25B | Ridge | East (A) | Yes | -10.4294 | 129.6671 | -10.4298 | 129.6671 |
| 28B | Ridge | East (A) | Yes | -10.3006 | 129.6499 | -10.3011 | 129.6497 |
| 35B | Terrace | East (A) | Yes | -10.4634 | 129.5389 | -10.464 | 129.539 |
| 44B | Terrace | East (A) | Yes | -10.4522 | 129.5242 | -10.4525 | 129.5248 |
| 45B | Terrace | East (A) | Yes | -10.5193 | 129.5227 | -10.5197 | 129.5236 |
| 10A | Valley | East (A) | Yes | -10.4303 | 129.656 | -10.4296 | 129.6567 |
| 11A | Valley | East (A) | Yes | -10.4428 | 129.554 | -10.4443 | 129.5539 |
| 12A | Valley | East (A) | Yes | -10.3737 | 129.5547 | -10.3727 | 129.5554 |
| 13A | Valley | East (A) | No | -10.3065 | 129.5595 | -10.3067 | 129.5585 |
| 26B | Valley | East (A) | No | -10.424 | 129.6201 | -10.4273 | 129.6192 |
| 32B | Valley | East (A) | Yes | -10.3014 | 129.5555 | -10.3002 | 129.5551 |
| 36B | Valley | East (A) | Yes | -10.4225 | 129.5864 | -10.4236 | 129.5864 |
| 46B | Valley | East (A) | Yes | -10.3716 | 129.5259 | -10.3717 | 129.5264 |
| 48B | Valley | East (A) | Yes | -10.393 | 129.4904 | -10.3929 | 129.4902 |
| 53B | Valley | East (A) | Yes | -10.5516 | 129.4801 | -10.5516 | 129.4806 |
| 21A | Bank | East (B) | Yes | -11.1429 | 129.9151 | -11.1433 | 129.9162 |
| 64B | Bank | East (B) | Yes | -11.0033 | 129.7842 | -11.0034 | 129.7844 |
| 65B | Bank | East (B) | Yes | -11.0391 | 129.8102 | -11.0389 | 129.8105 |
| 73B | Bank | East (B) | Yes | -11.1249 | 129.914 | -11.1253 | 129.914 |
| 26A | Plain | East (B) | Yes | -11.0894 | 129.8695 | -11.0876 | 129.8699 |
| 67B | Plain | East (B) | Yes | -11.0109 | 129.9074 | -11.0109 | 129.9078 |
| 22A | Ridge | East (B) | Yes | -11.008 | 129.7853 | -11.0069 | 129.7847 |
| 23A | Ridge | East (B) | Yes | -11.0377 | 129.8119 | -11.0365 | 129.8118 |
| 25A | Ridge | East (B) | Yes | -11.1629 | 129.826 | -11.1608 | 129.8259 |
| 1A | Bank | East (C) | Yes | -11.6353 | 129.8437 | -11.6364 | 129.8436 |
| 2A | Bank | East (C) | Yes | -11.5933 | 129.8248 | -11.5929 | 129.826 |
| 33A | Bank | East (C) | Yes | -11.6506 | 129.8314 | -11.65 | 129.8315 |
| 34A | Bank | East (C) | Yes | -11.6231 | 129.8313 | -11.623 | 129.8324 |
| 41A | Bank | East (C) | Yes | -11.6223 | 129.8354 | -11.6227 | 129.834 |
| 81B | Bank | East (C) | Yes | -11.621 | 129.8394 | -11.6208 | 129.84 |
| 3A | Terrace | East (C) | Yes | -11.5739 | 129.8352 | -11.5736 | 129.8388 |
| 29A | Terrace | East (C) | No | -11.7091 | 129.8111 | -11.7093 | 129.8083 |
| 31A | Terrace | East (C) | Yes | -11.6354 | 129.8215 | -11.6362 | 129.8205 |
| 32A | Terrace | East (C) | Yes | -11.5441 | 129.8238 | -11.5427 | 129.8238 |
| 63A | Terrace | East (C) | Yes | -11.677 | 129.8217 | -11.6775 | 129.8229 |
| 82B | Terrace | East (C) | Yes | -11.545 | 129.8239 | -11.5452 | 129.8236 |
| 4A | Valley | East (C) | Yes | -11.5415 | 129.8504 | -11.5402 | 129.8494 |
| 35A | Valley | East (C) | Yes | -11.5416 | 129.8341 | -11.5416 | 129.8346 |
| 36A | Valley | East (C) | Yes | -11.5529 | 129.846 | -11.5519 | 129.8452 |
| 38A | Valley | East (C) | Yes | -11.7136 | 129.8419 | -11.7123 | 129.843 |
| 4B | Plain | East (D) | Yes | -12.406 | 129.9537 | -12.4056 | 129.9537 |
| 5B | Plain | East (D) | Yes | -12.4029 | 129.9629 | -12.4027 | 129.9627 |
| 7B | Plain | East (D) | Yes | -12.357 | 129.97 | -12.3568 | 129.9699 |
| 9B | Plain | East (D) | Yes | -12.3366 | 129.9527 | -12.3366 | 129.9522 |
| 15B | Plain | East (D) | No | -12.324 | 130.0339 | -12.3244 | 130.034 |
| 16B | Plain | East (D) | No | -12.3285 | 130.0497 | -12.3287 | 130.0495 |
| 17B | Plain | East (D) | Yes | -12.3425 | 130.0626 | -12.3431 | 130.0626 |
| 19B | Plain | East (D) | Yes | -12.3964 | 130.0467 | -12.3966 | 130.0471 |
| 42A | Plain | East (D) | Yes | -12.2888 | 129.9756 | -12.2921 | 129.9754 |
| 45A | Plain | East (D) | No | -12.3328 | 129.9536 | -12.3318 | 129.9535 |
| 46A | Plain | East (D) | No | -12.3577 | 129.953 | -12.3565 | 129.953 |
| 48A | Plain | East (D) | Yes | -12.4055 | 129.9688 | -12.4043 | 129.9688 |
| 49A | Plain | East (D) | Yes | -12.2916 | 130.0025 | -12.2904 | 130.0025 |
| 50A | Plain | East (D) | No | -12.3121 | 130.016 | -12.3105 | 130.0158 |
| 52A | Plain | East (D) | No | -12.3492 | 130.0228 | -12.3493 | 130.024 |
| 53A | Plain | East (D) | Yes | -12.3502 | 129.9686 | -12.3502 | 129.9676 |
| 54A | Plain | East (D) | No | -12.3583 | 129.988 | -12.3595 | 129.9881 |
| 56A | Plain | East (D) | Yes | -12.3855 | 130.0184 | -12.3853 | 130.0173 |
| 57A | Plain | East (D) | Yes | -12.4002 | 130.0535 | -12.3996 | 130.0523 |
| 6B | Valley | East (D) | Yes | -12.4031 | 129.9866 | -12.4032 | 129.9863 |
| 10B | Valley | East (D) | No | -12.3041 | 129.9812 | -12.3044 | 129.9813 |
| 11B | Valley | East (D) | Yes | -12.2975 | 129.9641 | -12.2972 | 129.9639 |
| 12B | Valley | East (D) | No | -12.2888 | 129.9537 | -12.2877 | 129.9537 |
| 13B | Valley | East (D) | Yes | -12.3158 | 129.9393 | -12.3161 | 129.9394 |
| 14B | Valley | East (D) | Yes | -12.3222 | 129.9538 | -12.3225 | 129.9541 |
| 18B | Valley | East (D) | Yes | -12.3167 | 130.0623 | -12.3172 | 130.0623 |
| 20B | Valley | East (D) | Yes | -12.2985 | 129.9665 | -12.2988 | 129.9646 |
| 43A | Valley | East (D) | Yes | -12.2896 | 129.9519 | -12.2907 | 129.953 |
| 44A | Valley | East (D) | No | -12.3244 | 129.9557 | -12.3241 | 129.9545 |
| 47A | Valley | East (D) | No | -12.3903 | 129.9784 | -12.3892 | 129.9783 |
| 51A | Valley | East (D) | No | -12.3383 | 129.9945 | -12.3385 | 129.9956 |
| 55A | Valley | East (D) | Yes | -12.3825 | 129.9908 | -12.382 | 129.9897 |
| 6C | Bank | West (1) | Yes | -12.2039 | 127.4323 | -12.2037 | 127.4327 |
| 14C | Bank | West (1) | Yes | -12.0692 | 127.4361 | -12.0696 | 127.4361 |
| 15C | Bank | West (1) | Yes | -12.0688 | 127.4275 | -12.0684 | 127.4279 |
| 16C | Bank | West (1) | Yes | -12.0801 | 127.4290 | -12.0803 | 127.4296 |
| 24C | Bank | West (2) | Yes | -11.8864 | 127.0971 | -11.8862 | 127.0964 |
| 25C | Bank | West (2) | Yes | -11.8920 | 127.0753 | -11.8920 | 127.0750 |
| 26C | Bank | West (2) | Yes | -11.8993 | 127.0981 | -11.8989 | 127.0979 |
| 29C | Bank | West (2) | Yes | -11.8990 | 127.1024 | -11.8989 | 127.1027 |
| 31C | Bank | West (2) | Yes | -11.7945 | 126.9860 | -11.7949 | 126.9856 |
| 36C | Bank | West (2) | Yes | -11.8168 | 127.0438 | -11.8165 | 127.0441 |
| 37C | Bank | West (2) | Yes | -11.8188 | 127.0310 | -11.8182 | 127.0314 |
| 38C | Bank | West (2) | No | -11.8097 | 126.9699 | -11.8096 | 126.9706 |
| 46C | Bank | West (3) | Yes | -11.4335 | 126.9375 | -11.4330 | 126.9371 |
| 47C | Bank | West (3) | No | -11.4119 | 126.9491 | -11.4116 | 126.9493 |
| 53C | Bank | West (3) | Yes | -11.4405 | 126.9657 | -11.4399 | 126.9653 |
| 55C | Bank | West (3) | Yes | -11.4395 | 126.9513 | -11.4400 | 126.9519 |
| 56C | Bank | West (3) | Yes | -11.4015 | 127.0263 | -11.4015 | 127.0257 |
| 61C | Bank | West (3) | Yes | -11.4385 | 126.9684 | -11.4393 | 126.9682 |
| 63C | Bank | West (3) | Yes | -11.3972 | 126.9083 | -11.3969 | 126.9078 |
| 73C | Bank | West (1) | Yes | -12.0683 | 127.4276 | -12.0688 | 127.4269 |
| 74C | Bank | West (1) | Yes | -12.0748 | 127.4435 | -12.0754 | 127.4437 |
